# The Epidemiology of Injuries and Illnesses During SailGP Season 4: A Prospective Cohort Study

**DOI:** 10.3390/sports13030069

**Published:** 2025-02-27

**Authors:** Thomas Fallon, Hélène Rousselon, Neil Heron

**Affiliations:** 1 Centre for Public Health, Queen’s University Belfast, Belfast BT7 1NN, UK; tfallon02@qub.ac.uk; 2 Edinburgh Sports Medicine Research Network, UK Collaborating Centre on Injury and Illness Prevention in Sport (UKCCIIS), Institute for Sport, PE and Health Sciences, University of Edinburgh, Edinburgh EH8 9YL, UK; 3 Sail GP Medical Department, 5th Floor, London SW7 4ES, UK; helenerousselon@gmail.com

**Keywords:** sailing, Sports & Exercise Medicine, injury, illness

## Abstract

Introduction: Sailing is a high-intensity sport that demands a combination of physical strength, endurance, and technical skill. Sail Grand Prix (SailGP), inaugurated in 2019, epitomises a transformative approach to professional sailing. This study aims to provide a comprehensive analysis of injury and illness epidemiology among SailGP elite sailors, comparing injury and illness types and locations across different sailing activities. Methods: This prospective cohort study analysed injury and illness data from professional sailors throughout season 4 (2023/2024) of the professional sailing league. Data were collected on the number of hours spent in training, racing, and foiling, alongside injury reports, categorising injury types and locations. Incidence rates were calculated per 1000 h of sailing and 365 athlete days. Injuries were stratified by sex, position on the boat, and specific sailing activities to provide a detailed comparison. Results: A total of 100 sailors participated in the Sail GP 2024 season, accumulating 4919.84 h of recorded sailing activity: 2186.03 h in racing and 2733.80 h in training. The overall injury incidence rate was 9.96 per 1000 h of activity (95% CI: 7.17–12.75) and 7.43 per 365 athlete days (95% CI: 4.99–9.86). The injury rate was notably higher for foiling at 26.52 per 1000 h (95% CI: 19.09–33.94) compared to general sailing at 14.51 per 1000 h (95% CI: 10.44–18.57). Racing posed a greater injury risk than training, with an injury rate of 11.89 per 1000 h (95% CI: 7.77–16.46) compared to 8.41 per 1000 h for training (95% CI: 5.12–12.07). Grinders had the greatest rate of injuries at 3.86 (2.24, 5.69) injuries per 1000 h of sailing. The illness incidence rate was 5.14 per 1000 h (95% CI: 3.21–7.28), with respiratory illnesses being the most frequently reported. Conclusion: This study highlights the injury and illness risks amongst elite sailors in Sail GP. Males were at higher risk of injury, with the position of grinders being the most commonly injured. Ankle and knee injuries were the most prevalent. Future research should focus on developing sport-specific injury prevention programmes and examining long-term outcomes in sailors.

## 1. Introduction

**Figure 1:**
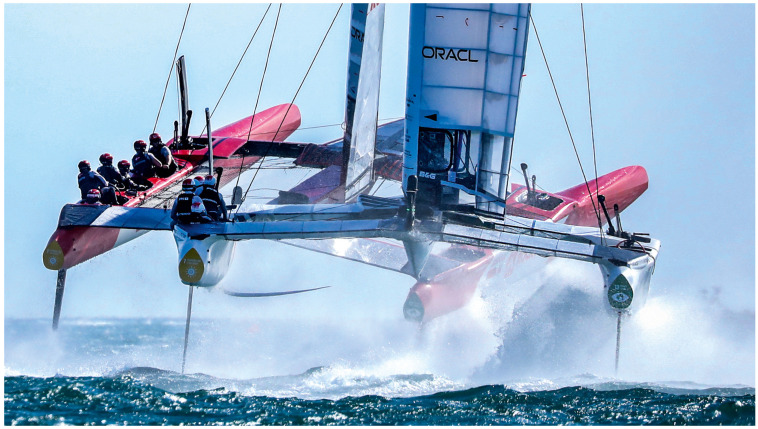
Two F50 foiling catamarans.

Sailing is a dynamic and multifaceted sport that entails the navigation of boats using sails, with a worldwide participation of approximately 16 million people [1]. Sailing encompasses diverse disciplines, competing from Olympic classes to commercial racing, each presenting unique challenges that test the limits of human performance and resilience. Sail Grand Prix (SailGP), inaugurated in 2019, epitomises a transformative approach to professional sailing. It serves as a global championship that showcases high-performance sailing, featuring cutting-edge technology and elite teams from across the globe racing F50 foiling catamarans that can reach speeds of 100 km/h (Figure 1). Foiling, as a type of sailing, is something that has appeared more widely in sailing in the last five to ten years. Foiling sailing differs from traditional sailing by using hydrofoils to lift the hull out of the water, drastically reducing drag and increasing speed. Unlike conventional boats that rely on hull and keel stability, foiling demands precise balance and constant adjustments to maintain flight. The high speed and dynamic demands of this type of racing require athleticism, teamwork, and tactical intelligence from the sailors and support staff. SailGP has positioned itself as a leader in advocating for sustainable practices in sports, promoting a vision of a cleaner and more sustainable future for both the sport and the planet.

There is a paucity of injury and illness epidemiological research in sailing [2]. The injury rates among Olympic-level sailors during competition have been reported to be between 0.2 and 0.59 injuries per year [3,4,5,6]. To date, only one study has prospectively documented the injury and illness rates among elite sailors [7]. It was found that the injury incident rate was 3.71 injuries per year, with no difference between the sexes. The main injury region was the lumbar spine, followed by the knee and ankle. Muscle and tendon injuries were the most common injury types, which is in line with previous research [2,3,7]. It has been identified that specific movements, such as hiking, may increase the risk of injury; however, among the F50 boats used within SailGP, these movements are not required.

Illness epidemiology in sailing has been mainly conducted based on retrospective reports [3] or within competitions [4,5,6], which show that sailing has one of the highest rates of illness among all Olympic sports. Most illnesses are reported to involve the respiratory system, followed by the gastrointestinal system [3,4,5,6]. To date, only one prospective study described illness rates among sailors throughout the year [7]. Respiratory infections accounted for 40.7% of all illnesses reported, with an illness rate of 0.57 (95%CI = 0.43–0.75) illnesses per 365 athlete days. Females were found to have a 3.6-fold increase in illnesses compared to males (Risk Ratio (RR) = 3.6; 95% CI = 2.0–6.7) [7].

As with any high-performance sport, the fast-paced, worldwide environment of SailGP exposes athletes to significant risks of injury and illness [2]. Understanding the epidemiology of these injuries and illnesses is crucial for developing effective prevention strategies [8,9]. Injury and illness epidemiology investigates the distribution, determinants, and outcomes of health-related events within a defined population. In the context of SailGP, this involves examining the frequency and types of injuries sustained during both training and competition, along with identifying contributory factors, such as environmental conditions, boat dynamics (foiling, various speeds), and the physical demands imposed on athletes. A unique aspect of SailGP is that males and females compete alongside each other. For the development of targeted, effective prevention strategies to occur, systematic injury and illness surveillance is paramount to enabling sports medicine clinicians to create the most evidence-based recommendations [8,10]. As SailGP continues to ascend in prominence and influence within the sporting realm, a thorough understanding of the epidemiology of injuries and illnesses in this context is essential. This study aims to prospectively quantify the incidence and prevalence of injuries and illnesses among athletes participating in SailGP. It is hypothesised that SailGP sailors experience a higher incidence of injuries during racing and foiling compared to training and general sailing, with grinders being at the highest risk due to their physically demanding role. This study will enable us to identify the most prevalent injuries and illnesses, as well as the associated risk factors, and facilitate the development of preventative strategies.

## 2. Methods

### 2.1. Study Design and Participants 

This prospective study was carried out between November 2023 and August 2024 following the professional sailing league SailGP. The participants of this prospective, observational study were male and female sailors competing in the professional SailGP league. Ethical approval for this study was obtained from the Medical Faculty at Queen University Belfast, Northern Ireland (Faculty REC Reference Number MHLS 24_129—Amendment 1).

**Figure 2:**
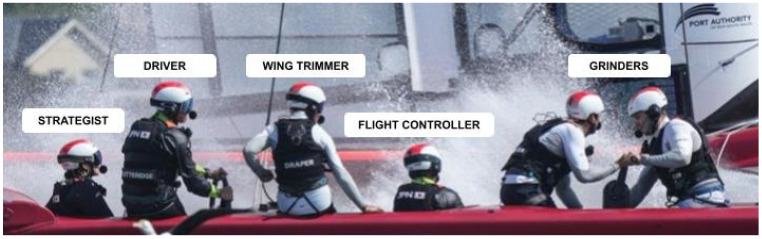
Sailors’ positions and roles.

On the F50 sailing boats, each position plays a crucial role in the operation of the high-performance vessel. Each boat has five male athletes and one female athlete, whose positions are broken down as follows (Figure 2):

1. *Grinders*: The only position on the boat that has two sailors. Responsible for generating hydraulic power through manual cranking to operate the sail and wing control systems.
2. *Flight Controller*: Manages the hydrofoils to maintain optimal lift and stability. This role is essential for controlling the height at which the boat flies above the water, impacting speed and manoeuvrability.
3. *Wing Trimmer*: Adjusts the wing sail’s angle and curvature to maximise aerodynamic efficiency. This position requires quick responses to varying wind conditions to ensure optimal speed.
4. *Driver*: Serves as the primary navigator and leader, steering the vessel and making strategic decisions. The skipper coordinates closely with the flight controller and wing trimmer to optimise performance during the race.
5. *Strategist*: Analyses racecourse and wind conditions to inform tactical decisions. The strategist collaborates with the skipper to develop and execute a competitive racing strategy.

### 2.2. Injury and Illness Definitions

Injury and illness definitions aligned with the IOC consensus statement [11]. Injuries were defined as “tissue damage or other derangement of normal physical function due to participation in sports, resulting from rapid or repetitive transfer of kinetic energy requiring medical attention.”

Illness was defined as “a complaint or disorder experienced by an athlete, not related to injury. Illnesses include health-related problems in physical (e.g., influenza), mental (e.g., depression) or social wellbeing, or removal or loss of vital elements (air, water, warmth).” All injuries and/or illnesses were diagnosed by a clinical professional (physiotherapist or doctor).

### 2.3. Data Collection

The event medical teams were required to complete all injury/illness forms daily through an online Qualtrics survey-based application and return them to the chief medical officer (CMO) for the league on the same day.The CMO screened the data daily, and any incomplete or unclear data were queried with the named person completing the form to allow full data capture. The questionnaire was developed from the IOC consensus on reporting and recording injuries and injury and illness diagnoses classified by the Orchard Sports Injury Classification System (OSICS) version 10.

### 2.4. Statistical Analysis

All data were processed on a Macintosh computer using Microsoft Office and R statistics (version 4.1.1; R Core Team 2014). The methods applied included frequencies (%), crosstabs, and descriptive statistics. All the sailors were analysed together, and the different demographics (gender and training vs. racing) were analysed separately to compare injury data between sexes, injury events (training/racing), injury types, and locations. All the statistical tests were two-sided, and results with *p* < 0.05 were statistically significant. 

### 2.5. Injury and Illness Incidence

To assess injury and illness incidence, incidence rates per 1000 h and 365 days were calculated. Data regarding total sailing time, boat time above 10 kph, and time foiling were provided by the league’s data insights team.

Total Sailing = Including “displacement mode” (Archimedean) (transits, breaks, waiting periods, or no-wind moments).

“Foiling” in sailing refers to the use of hydrofoils—wing-like structures attached to the bottom of a boat’s hull. As the boat gains speed, these hydrofoils lift the hull above the water surface, significantly reducing drag and allowing the boat to achieve much higher speeds than conventional sailing.

Further information on the number of training sessions, the number of racing sessions, total time training, and total time racing was also provided. Each team was assumed to have six sailors for both training and racing seasons. After determining competition sailing exposure (hours or days), the injury incidence was calculated using the number of on-water competition injuries identified from the online medical log (Equations (1) and (2)).

(1) $$Inj/1000\;h=\left(\frac{no.\;of\;inj}{no.\;of\;races\times 0.2\;h\;sailing\times no.\;of\;teams\times 6\;sailors}\right)\times 1000\;h$$

(2) $$Inj/365\;days=\left(\frac{no.\;of\;inj}{no.\;of\;days\;sailing\times no.\;of\;teams\times 6\;sailors}\right)\times 365\;days$$

## 3. Results

One hundred sailors took part in SailGP 2024 Season 4. The total time of recorded sailing activity was 4919.84 h, comprising 2186.03 h of racing and 2733.80 h of training. There were 229 training sessions and 224 racing sessions. The total time spent above ten kilometres per hour (kph) was 3378.16 h, including 1491.04 h for racing and 1887.12 h for training. Additionally, foiling accounted for 1848.69 h of sailing, with 814.90 h attributed to racing and 1033.79 h to training (Table 1).

### 3.1. Overall Injury Incidence Rates

There were a total of 49 injuries during the 2024 season. The mean age of injured individuals was 32.7 (SD 5.2) years. The mean age of females injured was 29.7 (SD 3.9) years, and the mean age of males was 33.6 (SD 5.2) years. The overall injury incidence rate was 9.96 per 1000 h of activity (95% CI: 7.17–12.75) and 7.43 per 365 athlete days (95% CI: 4.99–9.86). Specifically, the injury rate for foiling was markedly higher at 26.52 per 1000 h (95% CI: 19.09–33.94), compared to 14.51 per 1000 h for sailing (95% CI: 10.44–18.57) (Table 2).

Injury rates were higher during racing than during training, with 26 reported injuries in racing events, corresponding to a rate of 11.89 per 1000 h (95% CI: 7.77–16.46) and 3.95 per 365 athlete days (95% CI: 2.59–5.48). In contrast, training resulted in 23 injuries, with a rate of 8.41 per 1000 h (95% CI: 5.12–12.07) and 3.50 per 365 athlete days (95% CI: 2.13–5.02). Injury rates were significantly different between the sexes. Males had an injury rate of 7.52 per 1000 h (95% CI: 5.29–9.96) and 5.63 per 365 athlete days (95% CI: 3.95–7.45), while females had a lower rate of 2.44 per 1000 h (95% CI: 1.22–3.86) and 1.83 per 365 athlete days (95% CI: 0.91–2.89). Among different sailing positions, the highest injury rates were observed in the “Grinder” position, with 3.86 per 1000 h (95% CI: 2.24–5.69) and 2.89 per 365 athlete days (95% CI: 1.67–4.26) (Table 3).

Overall, ankle ligament injuries were the most common injury at 1.50 (95% CI: 0.42–2.78)/1000 h, followed by knee contusions at 1.07 (95% CI: 0.214–2.14)/1000. (Table 4) Male athletes most frequently experienced injuries to the ankle and knee, each with a rate of 1.22 per 1000 h. Female athletes also reported injuries to the ankle but at a lower frequency (0.81 per 1000 h). The most common types of injuries for both sexes were contusions and ligament injuries.

### 3.2. Illness Incidence Rates

The total number of reported illnesses was 24, leading to an overall illness incidence rate of 5.14 per 1000 h (95% CI: 3.21–7.28) and 3.65 per 365 athlete days (95% CI: 2.28–5.17). The overall illness burden per 1000 h was 2.35 (95% CI: 1.47–3.38). Illness incidence rates differed between sexes, with males reporting 19 illnesses (3.86 per 1000 h) and females reporting 5 illnesses (1.02 per 1000 h). Most illnesses reported were respiratory (13 cases), corresponding to an incidence rate of 2.64 per 1000 h (95% CI: 1.22–4.27), with a severity of 0.67 days lost per illness.(Table 5) Other reported illnesses included neurological, ophthalmological, vestibular, and gastrointestinal conditions.

## 4. Discussion

This is the first study to prospectively present injuries and illnesses across a SailGP season. This study follows on from a retrospective analysis of season 3 injuries and illness insights (under review—journal of *BMJOSEM*). The main points highlighted within this study of season 4 are as follows:

- The overall injury rate was 9.96 injuries per 1000 h and 7.43 per 365 days, with foiling having a significantly higher injury rate (26.52 per 1000 h) than general sailing (14.51 per 1000 h).
- Injury rates were higher during racing (11.89 injuries per 1000 h) compared to training (8.41 injuries per 1000 h), with similar trends in the rate per 365 athlete days.
- Males had a notably higher injury rate (7.52 per 1000 h) compared to females (2.44 per 1000 h), and this difference was also reflected in the rate per 365 days.
- The most common injury locations for males were the ankle and knee (both 1.22 per 1000 h), while for females, the ankle was most frequently injured (0.81 per 1000 h).
- Contusions were the most frequent injury type, particularly in males (1.83 per 1000 h), with ligament and muscular strains/spasms also common for males at 0.610 (0.000–1.420 per 1000 h)
- The overall illness rate was 5.14 per 1000 h and 3.65 per 365 athlete days, with respiratory illnesses being the most frequent (2.64 per 1000 h).

The overall injury rate was calculated at 9.96 injuries per 1000 h of activity, with a notable distinction between racing and training environments. The injury rate during racing was higher (11.89 per 1000 h) compared to training (8.41 per 1000 h). These rates of injury are lower than what has been seen in international rugby [12] union; however, they are higher than what has been seen in women’s football [13]. The overall injury rate is comparable to that in professional male professional football at 8.1 injuries/per 1000 h of exposure, with a match injury incidence of 36 injuries per 1000 h in matches [14]. Specific activities such as foiling demonstrated a significant injury incidence rate of 26.52 per 1000 h, which may be attributed to the higher speeds achieved during foiling rather than the activity itself. As the overall exposure hours included in this study are broken down into overall sail time, which includes tow time, time above 10 kph, and time foiling, the denominator of overall time will dilute the true injury risk. Using time above 10 kph and foiling time are more accurate representations of the injury rate within sailing. Recent advancements in Sail GP F50 boats are their new foiling technology. With this, improving athletes’ overall ability to transfer from each side of the boat during dynamic movements at speed is an important factor that should be incorporated into injury prevention initiatives of sailing early on. SailGP has specific boat simulators for training athletes to use the boats, helping to improve their familiarity with these new boats, particularly around activities such as foiling, which are higher risk with the higher speeds involved. It would also be prudent to consider developing robust injury prevention routines specific to Sail GP and to develop these programmes from injury/illness epidemiology work. These programmes could be developed similarly to the FIFA11+ [15] GAA 15 and Prep-to-Play PRO [16] injury prevention programmes [17], which have been shown to reduce injury risk within these specific sports. Such programmes have been shown to facilitate significant reductions in injury incidence, which would have some crossover with Sail GP given the high injury rates seen in athletes, predominantly in the lower limbs.

The most common athletes within the boat to sustain injuries were grinders, with an injury rate of 3.86 injuries (95% CI: 2.24, 5.69) per 1000 h. These athletes are responsible for generating power using their arms, which the wing trimmers then use to control the sail and generate boat speed. However, the grinder role is the only position on the boat that has two athletes, and thus, this injury rate is partly a reflection of having a higher number of athletes in this category and, therefore, a higher number of absolute injuries. Moreover, within the America’s Cup, the grinder role has evolved to using legs to generate power through a stationary bike type, which will arguably see greater power output and likely lower the injury risk associated with this boat position [18]. As the SailGP league evolves, this may be a consideration to improve the speed of boats and reduce injury rates within this specific position.

When comparing these rates with those in other sports, sailing presents unique challenges. In high-impact sports such as male football, the injury incidence can range from 3.7 to 36 injuries per 1000 h [14]. The trend of more injuries in matches (36/1000 h) compared to training (3.7/1000 h) is similar to what has been noted within sailing. However, the rates remain lower overall in SailGP. The most common injuries seen in sailing were to the lower limbs, particularly ligament injuries, at a rate of 1.50 (95% CI: 0.42–2.78)/1000 h. The injury locations and patterns of injury within sailing have similarities to those in field-based sports. Water-based sports such as SailGP have the additional factor of being held on water and thus the additional risk of drowning, necessitating immediate medical management of injuries/illness, particularly those involving loss of consciousness [17,19,20,21].

Although the precise role of meteorological data in injury prevention within SailGP has not yet been examined, incorporating such data into future analyses could prove valuable. Including variables like wind speed, water conditions, and other weather factors would enhance the understanding of environmental factors such as aggressive winds, changes in wind direction, and fluctuating water conditions, which can significantly impact the dynamics of sailing. By integrating meteorological data, future studies can better assess how these external conditions may contribute to injury risk during competitions. This approach would provide a more comprehensive understanding of the factors affecting performance and predisposing athletes to injuries. Subsequently, it will help develop more targeted injury prevention strategies tailored to varying weather conditions during competitions such as pre-race educational briefings for medical teams and athletes.

Illness rates, particularly respiratory illnesses, are frequently noted in endurance sports like marathon running, where athletes experience increased exposure to pathogens during competition. This presents an area of concern in sailing, particularly during events that expose athletes to varying weather conditions, which could lead to respiratory challenges. Additionally, the sport is global, with high travel demands being placed on athletes. To help reduce the impact of illness within Sail GP, athletes and coaching staff partaking in the league should be educated about hand hygiene, the use of disinfectants, mask-wearing, the avoidance of handshaking—especially with other teammates and staff—media, and social distancing (buffet environments, planes, bus) [19,20]. Furthermore, athletes should consider supplements such as zinc, vitamin D, and probiotics, which may help boost the immune system and reduce susceptibility [19,21]. Consideration should be given to the increased risk of pathogens on flights during travel. Mitigation measures such as mask-wearing, awareness of higher-risk seating positions on aircraft, and the limitation of movement around the cabin during flight should be given to all athletes before travel, with an additional emphasis on female and visually impaired athletes [19]. These findings are in line with recent IOC statements showing the risk of respiratory illness across all athletes [19,20].

In sailing, injuries are frequently reported on an informal basis, leading to gaps in data collection and analysis. Ours is the first study to present granular detail around injury and illness exposures. The ability to do this is achieved through collaboration with data scientists as part of the league. Further injury prevention studies should continue to develop working relationships with data analysis teams, as these insights can give injury and illness epidemiology research more granular insights into the rates of injuries and specific events/manoeuvres that pose greater risks. The lack of consistent historical data limits our ability to implement effective preventive strategies, potentially resulting in higher injury rates in the future. A more systematic approach to surveillance would involve developing standardised reporting forms and creating centralised databases to monitor trends over time [22]. This systematic approach to injury and illness prevention is well documented and has proven successful in other sports, where comprehensive injury data have informed rule changes, training modifications, and enhanced medical support [23,24,25,26,27].

### 4.1. Clinical Recommendations

To reduce injuries and illnesses in SailGP athletes, it is essential to prioritise strength and conditioning programmes, particularly focusing on core stability, ankle/knee proprioception, and upper-body strength for foiling and high-speed sailing. Neuromuscular and balance training should be implemented to prevent ankle and knee injuries, while scapular control and wrist/forearm strengthening exercises can help avoid upper-extremity overuse injuries in grinders. To minimise respiratory illnesses, optimal nutrition and stress management should be promoted, with further consideration given to supplements such as zinc, vitamin D, and probiotics, which may help boost the immune system and reduce susceptibility [22,24]. Lastly, given the global nature of the league, consideration should be given to the increased risk of pathogens on flights during travel.

### 4.2. Limitations

Although several limitations must be acknowledged, this study provides valuable insights into injury and illness epidemiology among professional SailGP sailors. First, this study focused exclusively on professional sailors participating in the SailGP league. A higher proportion of male participants was observed, which may result in an underestimation of injury and illness rates among female sailors. This discrepancy highlights a critical gap in the current literature, as women remain underrepresented in elite-level sailing. Future studies should aim to collect more robust data on female sailors to better understand their unique injury patterns, physiological demands, and health challenges. Addressing this imbalance will contribute to greater inclusivity and equity within the sport and enhance our understanding of sex-specific risk factors.

Second, we relied on team-wide navigation times to calculate exposure instead of individual sailor hours. This introduces a potential bias: sailors with higher workloads (e.g., more active roles or greater time on the water) are likely to have a higher risk of injury compared to those with reduced exposure. Conversely, less active sailors—due to rotation, rest periods, or role-specific tasks—may artificially lower the overall injury rate. This lack of granular, individual-level exposure data limits the precision of our incidence rate calculations and may obscure individual variations in injury risk. Future research should explore wearable technology or tracking systems to record individual athlete exposure in real time, improving data accuracy.

Third, for simplicity, we assumed that all boats were operated with six sailors onboard at all times. However, we know that during certain training or racing sessions, the teams may have sailed with fewer crew members due to race rule decisions. This assumption could inflate the denominator for exposure hours, leading to a slight underestimation of injury and illness incidence rates. Future studies should aim to integrate session-specific crew data to provide a more accurate calculation of exposure and incidence.

Finally, the data were collected exclusively over a single SailGP season. While this provides a valuable snapshot, inter-seasonal variations—such as changing environmental conditions, boat technology advancements, or athlete conditioning programmes—may influence injury and illness patterns. Longer-term, multi-season studies are needed to identify trends over time, assess the effectiveness of prevention programmes, and explore cumulative risks, particularly for chronic injuries.

## 5. Conclusions

**Figure 3:**
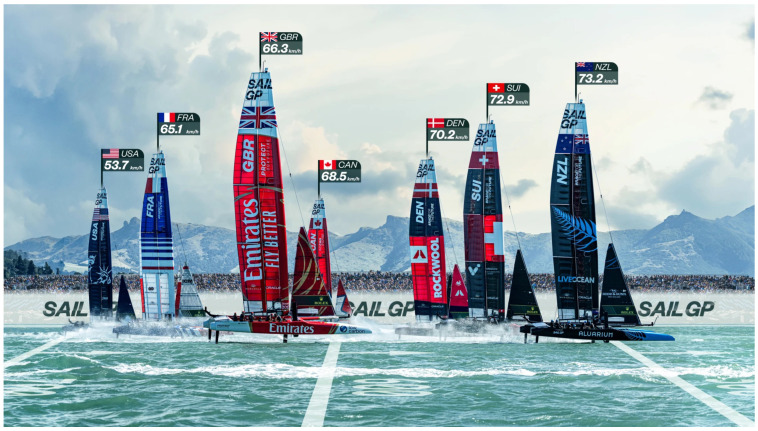
Boats on water.

This study provides a comprehensive epidemiological analysis of injuries and illnesses in SailGP Season 4, identifying critical areas for intervention to enhance athlete safety. (Figure 3) The high injury incidence during foiling and racing highlights the urgent need for sport-specific prevention programmes, particularly targeting lower-limb stability, upper-body strength, and core control. Given the elevated rates of respiratory illnesses, implementing targeted education, enhanced hygiene protocols, and travel-based precautions is essential.

To further mitigate risks, SailGP organisers should consider integrating mandatory strength and conditioning programmes tailored to different athlete roles and utilising off-water simulations that recreate high-speed manoeuvres. Additionally, the observed sex-based disparities in injury rates underscore the importance of gender-specific research to better understand risk factors and optimise training and prevention strategies for all athletes. As SailGP continues to evolve, leveraging data-driven insights with proactive safety measures will be crucial in reducing injury burdens, improving athlete performance, and ensuring long-term participation in professional sailing.

**What is already known on this topic**
  - Previous research has established that professional athletes in high-performance sports face significant risks of injuries and illnesses, with certain roles and activities (e.g., high-speed or impact-based events) carrying higher risks. However, injury rates in water-based sports, particularly professional sailing, are less well documented compared to land-based sports such as rugby or football.
  - Recent advancements in sailing technology, such as hydrofoils, have introduced higher speeds and additional physical demands, which likely increase injury risks. Limited data exist on how these innovations affect injury patterns, necessitating further study.
**What this study adds**
  - This study is the first to prospectively quantify injury and illness rates in a professional sailing league (SailGP), establishing overall rates of 9.96 injuries per 1000 h and 5.14 illnesses per 1000 h. It highlights significant differences between racing and training environments, as well as the elevated risk of foiling (26.52 injuries per 1000 h).
  - Grinders experienced the highest injury rates among all positions, with lower-limb injuries (e.g., ankle ligaments) being the most common. Males exhibited significantly higher injury rates than females, reflecting possible differences in physical demands or exposure patterns.
  - Respiratory illnesses were the most common, with global travel, varying weather conditions, and team dynamics identified as potential risk factors. This study also quantified the illness burden, shedding light on recovery times and impacts.
**How this study might affect research, practice, or policy**
  - The findings emphasise the need for role-specific injury prevention programmes, particularly for grinders, and strategies addressing foiling-related risks. These programmes could adapt elements from successful initiatives in other sports, such as FIFA 11+ or Prep-to-Play PRO.
  - SailGP organisations may need to enforce stricter safety protocols during racing and foiling activities, including the mandatory use of advanced simulators and enhanced personal protective equipment to mitigate risks.
  - The study’s identification of illness patterns could inform health policies, including travel management, hygiene protocols, and nutritional supplementation strategies (e.g., zinc, vitamin D, probiotics) to reduce illness incidence during global sailing events.

## Figures and Tables

**Table 1:** Total sailing time.

Activity Summary	Overall (h)	Racing (h)	Training (h)
Time above 10 kph	3378.16	1491.04	1887.12
Time foiling	1848.69	814.90	1033.79
Total sailing time	4919.84	2186.03	2733.80

**Table 2:** Overall injury incidence rates (95% Confidence Interval).

Overall Injury Incidence Rates	Rate (95% CI)
Overall injury rate per 1000 h	9.96 (7.17–12.75)
Overall injury rate per 365 days	7.43 (4.99–9.86)
Injury rate for foiling per 1000 h	26.52 (19.09–33.94)
Injury rate for sailing >10 kph per 1000 h	14.51 (10.44–18.57)

**Table 3:** Injury incidence rates: training vs. racing, sex, and boat position (95% Confidence Interval).

Event	Rate Per 1000 h (95% CI)	Rate Per 365 Athlete Days (95% CI)
Training	8.41 (5.12–12.07)	3.50 (2.13–5.02)
Racing	11.89 (7.77–16.46)	3.95 (2.59–5.48)
**Sex**	**Rate Per 1000 h (95% CI)**	**Rate Per 365 Athlete Days (95% CI)**
Male	7.52 (5.29, 9.96)	5.63 (3.95, 7.45)
Female	2.44 (1.22, 3.86)	1.83 (0.91, 2.89)
**Position**	**Rate Per 1000 h (95% CI)**	**Rate Per 365 Athlete Days (95% CI)**
Grinder	3.86 (2.24, 5.69)	2.89 (1.67, 4.26)
Flight controller	1.42 (0.41, 2.64)	1.06 (0.30, 1.98)
Wing trimmer	1.63 (0.61, 2.85)	1.22 (0.46, 2.13)
Strategist	1.83 (0.81, 3.05)	1.37 (0.61, 2.28)
Driver	1.22 (0.41, 2.24)	0.91 (0.30, 1.67)

**Table 4:** Summary of injuries by location and type (95% Confidence Interval).

Type	Location	Sex	Count	Rate Per 1000 h (95% CI)	Rate Per 365 Athlete Days (95% CI)
Ligament	Ankle	F	4	0.813 (0.203, 1.630)	0.608 (0.152, 1.220)
Ligament	Ankle	M	3	0.610 (0.000, 1.420)	0.456 (0.000, 1.060)
Muscular Spasm	Lower Back	M	3	0.610 (0.000, 1.420)	0.456 (0.000, 1.060)
Contusion	Knee	M	3	0.610 (0.000, 1.420)	0.456 (0.000, 1.060)
Jet Lag	Head	M	2	0.407 (0.000, 1.020)	0.304 (0.000, 0.760)

**Table 5:** Illness incidence rates by system (95% Confidence Interval).

System	Rate Per 1000 h (95% CI)	Rate Per 365 Athlete Days (95% CI)
Respiratory	2.64 (1.22, 4.27)	1.96 (0.92, 3.18)
Neurological	0.41 (0.000, 1.020)	0.30 (0.000, 0.760)
Ophthalmological	0.41 (0.000, 1.020)	0.30 (0.000, 0.760)
Gastrointestinal	0.61 (0.000, 1.420)	0.46 (0.000, 1.060)

## Data Availability

The data that support the findings of this study are available from the corresponding author upon reasonable request.
